# Jailed or freed? The lead dilemma in transcatheter tricuspid valve replacement

**DOI:** 10.1093/europace/euaf235

**Published:** 2025-09-23

**Authors:** Pascal Defaye, Peggy Jacon, Sandrine Venier

**Affiliations:** Cardiology Department, University Hospital and Grenoble Alpes University, Grenoble, France; Cardiology Department, University Hospital and Grenoble Alpes University, Grenoble, France; Cardiology Department, University Hospital and Grenoble Alpes University, Grenoble, France

**This editorial refers to ‘Device-lead abnormalities and function after transcatheter tricuspid valve replacement’ by M. Abbasi *et al.*, https://doi.org/10.1093/europace/euaf219.**

## Introduction

Transcatheter tricuspid valve replacement (TTVR) has rapidly emerged as a promising alternative for patients with severe tricuspid regurgitation (TR) deemed high risk for surgery. Yet, a significant number of these patients have cardiac implantable electronic devices (CIEDs) often the very cause or aggravator of TR. A recent meta-analysis confirmed that right ventricular pacing via trans-tricuspid leads significantly increases the risk of TR, whereas conduction system pacing (CSP) and leadless options were more favourable.^1^

Among available technologies, the EVOQUE system has shown promising outcomes. In the TRISCEND II trial, 38.2% of patients treated with Evoque had a CIED lead at baseline with a conservative approach.^2^ The strategy of jailing leads during TTVR is traditionally discouraged due to risks of mechanical dysfunction and the potential impossibility of future extraction in case of infection or system failure. Consequently, the management of TTVR remains a critical and unresolved challenge.

The study by Abbasi *et al.* provides essential insights into the consequences of leaving pacemaker and ICD leads in place during TTVR the so-called ‘jailed leads strategy’.^3^

In this cohort of 32 patients undergoing EVOQUE implantation with preexisting CIED lead crossing the tricuspid valve and left *in situ*. At a median follow-up of 210 days, 31% developed lead abnormalities, including increased capture threshold, sensing alterations, or even lead failure requiring revision. This is the first study to quantify and characterize these complications retrospectively, raising crucial questions about optimal lead management. These findings challenge earlier reports, which may have under-recognized lead complications due to limited sample sizes and the absence of systematic lead monitoring.^4^ Supporting these concerns, Mekary *et al.* reported severe lead dysfunction in 20% of patients, including two deaths (one sudden), and complications after extraction of a jailed lead.^5^

Some authors have advocated a selective extraction strategy, for example, in pacemaker-dependent patients or in ICD leads with appropriate therapies but this approach remains highly debatable.^6^

## The clinical dilemma: jailing vs. extraction

When a CIED lead crosses the tricuspid annulus in a candidate for EVOQUE TTVR, the decision is rarely straightforward. When feasible, expert consensus currently favours transvenous lead extraction (TLE) before TTVR to avoid post-procedural dysfunction and to optimize valve deployment.^7,8^ The recent European scientific statement explicitly recommends avoiding jailed leads in several scenarios: pacemaker dependency, ICDs with prior therapies, multiple transvalvular leads, history of CIED infection, multiple infection risk factors, or high lead tension.^7^ However, these recommendations are largely based on expert opinion and low-level evidence, reflecting the current lack of high-quality data or randomized trials. Consequently, real-world practice often requires individualized decisions, guided more by clinical judgment and local expertise than by prescriptive guidelines.

Jailed leads may be subjected to high mechanical stress, impingement, or interference with the prosthetic valve stent frame, resulting in fracture, dysfunction, or infection. Thus, pre-procedural TLE should be considered.^9^

However, TLE carries its own hazards. In frail patients with advanced right heart failure, extraction is risky, especially when leads are old or adherent. Extraction may destabilize valve anatomy, exacerbate TR, or even preclude TTVR. Complications may include paravalvular leak, embolization of vegetations or lead fragments, annular damage, or lead fracture.^10^ Clinicians must balance procedural feasibility, device preservation, and long-term safety.

The Abbasi study highlighted that 23.7% of jailed leads developed electrical abnormalities and 6.8% mechanical damage requiring intervention.^3^ Dysfunction was not confined to older leads or ICD leads; pacemaker leads were also affected. Imaging suggests that impingement by the EVOQUE frame, especially with horizontal RV lead orientation, is a key mechanism. Long-term consequences remain unknown, but progressive fibrosis and stent–lead interaction may worsen dysfunction over time.

## A multidisciplinary imperative

A truly *multidisciplinary ‘heart team’* approach is crucial in navigating the complex interplay between structural intervention and device management.^10^ In this setting, pre-procedural planning must involve electrophysiologists with TLE expertise, interventional cardiologists, anaesthetists, imaging specialists, and cardiac surgeons. The ‘heart team’ concept is already established in TTVR management and particularly indispensable when addressing lead-related decision.^10,11^

## Management options beyond jailing

Lead extraction with reimplantation, either via coronary sinus or leadless pacing, is an appealing option.

This approach eliminates the risk of impingement but requires high-volume TLE expertise. Leadless devices (e.g. Micra™, Aveir™) are feasible and safe post-tricuspid interventions.^12^ Coronary sinus pacing is also a possible alternative but can be technically challenging post-EVOQUE due to the prosthesis bulk, anatomical variability of the venous system, and pacing thresholds. Furthermore, it is only compatible with selected pacemaker systems. Surgical epicardial pacing is now largely obsolete.

Newer options such as dual-chamber leadless pacemaker (LP) or even fully leadless CRT may offer promising long-term solutions.^13^ In the future, CSP using leadless technology is also expected to become available.

## Future directions/perspectives

The retrospective nature of existing data and the short duration of follow-up in most cohorts limit our ability to predict long-term lead performance and valve interaction. The Abbasi study raises key unanswered questions:

- **Long-term durability**: Will early lead dysfunction progress, leading to fractures or capture loss? Data beyond 210 days are lacking. What are the implications if extraction of a jailed lead becomes necessary due to infection? Could removal destabilize the EVOQUE valve and cause migration?
- **Pre-procedural imaging**: Can imaging predict patients at high risk for lead dysfunction, particularly based on commissural positioning?
- **Role of leadless systems**: With the advent of dual-chamber LP and emerging leadless CRT systems,^14,15^ should pre- or post-TTVR leadless implantation become standard in high-risk patients?

The presence of an existing LP may also pose technical challenges during TTVR, including potential interactions and procedural interference, as well as the potential difficulties of LP implantation as experience expands to a larger cohort of patients undergoing TTVR.

Prospective registries and possibly randomized trials comparing lead management strategies in TTVR are urgently needed.

## Conclusion

Current evidence shows that jailed leads during EVOQUE TTVR carry a substantial risk of dysfunction. Although pre-emptive extraction entails procedural risks, the strategy of leaving leads in place should no longer be considered benign or neutral. The decision must balance patient frailty, comorbidities, lead age, number, echocardiographic lead–valve relationship, and device type.

Where feasible, expert consensus supports pre-procedural extraction, ideally with reimplantation using leadless systems.^7^ Yet, for many patients with old or fragile leads, extraction may not be an option. In such cases, individualized heart team discussion and careful follow-up with remote monitoring are essential. Post-TTVR reimplantation strategies, preferably using leadless pacing should be systematically considered and performed in high-expertise centres with equal competence in device and interventional cardiology.
